# Personalized Treatments for Functional Disorders of the Sphincter of Oddi: A Short Muscle with a Long History of Discussion and Controversies

**DOI:** 10.3390/jpm16020106

**Published:** 2026-02-10

**Authors:** Zoltán Berger, Ákos Pap

**Affiliations:** 1 Departamento Medicina, Hospital Clínico Universidad de Chile, Carlos Lorca Tobar 999, Independencia, Santiago 8320000, Chile; 2 Clinica Las Condes, Estoril 450, Las Condes, Santiago 8380456, Chile; 3 Institute of Pancreatic Diseases, Semmelweiss University, Tömö u. 25-29, 1083 Budapest, Hungary

**Keywords:** sphincter of Oddi, bile duct, pancreatic duct, spontaneous passage of bile duct stones, hypertonic dyskinesia, diagnostic tests, pharmacologic treatment, endoscopic sphincterotomy

## Abstract

The sphincter of Oddi (OS) is a small group of smooth muscles that plays a crucial role in the regulation of the flow of biliopancreatic secretions into the duodenal lumen. Its motility, including phasic contractions and relaxation, is under complex neurohumoral control. Organic or functional obstruction of the OS is an important factor in severe diseases, such as cholangitis and pancreatitis, as well as in functional disorders and recurrent abdominal pain. In this review, we summarize the function of the OS, its disorders, and their diagnostic methods and potential therapeutics. While organic diseases of the papilla often require invasive, mainly endoscopic, treatment, functional disorders should be managed with conservative, individualized treatment, and involve the patient and their family in decision-making.

## 1. Introduction

### 1.1. Physiology

The sphincter of Oddi (OS), first described by Ruggero Oddi [1,2], comprises a small group of smooth muscles that regulate the flow of biliopancreatic secretions into the duodenum, making it one of the most important small organs in the human body. The proper sphincter of Oddi is located where the common biliopancreatic channel enters the duodenal wall, while two smaller groups are located before the union of the two ducts: the sphincter of the choledochus and the pancreatic sphincter. These muscle groups function in a synchronized manner; but disorders of the OS do not always involve all these groups and can separately affect emptying of bile or the pancreatic secretion.

Relaxation of this muscle permits the efficient mixing of bile and pancreatic enzymes with food in the duodenal lumen and its contractions in interdigestive phases prevent the reflux of duodenal contents into the bile and pancreatic ducts. Thus, this small sphincter has important physiological functions; however, its dysfunction and the consequences have been a subject of debate.

Complex neurohumoral regulation ensures relaxation of the OS in the presence of food in the duodenum. Cholecystokinin (CCK) is the principal hormone in this regulatory process. It is released from the duodenal and jejunal mucosa, and functions to simultaneously increase biliopancreatic secretion and relax the OS [3]. These functions are mainly mediated through neural pathways: CCK-A (type 1.) receptors only recently were detected in human pancreatic acinar cells [4,5] and CCK induces contraction of isolated OS muscle [6], the OS relaxation requires integrity of duodenum and neural fibers [7]. Morphine and other opiates induce OS contraction. Other drugs can relax the OS including nitric oxide (NO) donors, (nitrates being the most efficient) [8], certain calcium antagonists [9], and phosphodiesterase inhibitors, such as aminophylline [10], theophylline [11], and vardenafil [12]. OS dysfunction has been linked to several pathological conditions, for example, exaggerated OS contractions and high OS pressure have been associated with bilio-pancreatic pain, cholangitis [13], and recurrent acute pancreatitis (RAP) [14]. The absence of sphincter function after surgical or endoscopic ablation of the OS may facilitate duodenobiliary or duodenopancreatic reflux, bacterial contamination, and biliary stone formation [15,16]. A rare case of a hypotonic OS was described, which reported biliary type abdominal pain accompanied by pneumobilia and emphysema of the pancreatic duct [17]. In clinical practice, OS functional disorders are frequently suspected when there are no objective structural changes to the OS that can explain the patient’s symptoms. However, excluding other organic disorders is challenging and objective and reproducible diagnostic methods for sphincter of Oddi dyskinesia (SOD) are lacking. Consequently, even the existence of this functional disorder is frequently questioned [18].

In this paper, we describe our personal experiences with OS disorders and a provide a review of the recent literature on the OS’s role in pathological conditions and potential treatments, with a particular emphasis on the different treatment modalities for SOD and their risks vs. benefits.

### 1.2. Pathology

#### 1.2.1. Gallstone Passage and the OS

Cholelithiasis is a globally prevalent pathology. Gallstones usually pass from the gallbladder to the bile duct and finally into the duodenal lumen through the papilla of Vater [19,20]. However, rare complications can occur where biliary obstruction results in jaundice with or without cholangitis, or temporary obstruction of pancreatic outflow leads to pancreatitis. The prevalence of these complications remains unknown, as do the factors that increase their risk. Bile stones are the dominant etiology for AP in most of the world [21,22,23,24,25]. However, a stone only causes AP if it is impacted in the papilla or damages the papillary orifice during its passage. Acosta and Ledesma demonstrated spontaneous stone passage in the majority of patients with biliary pancreatitis [26]. Acosta also demonstrated a clear correlation between the duration of ampullary obstruction and the severity of pancreatic damage [27]: a more benign, frequently uncomplicated evolution was observed if the ampullary obstruction was resolved spontaneously or via ERCP within 48 h. Currently, urgent ERCP with papillotomy and stone extraction is only considered necessary in cases of pancreatitis associated with cholangitis and impacted stones [28] and no clinical utility was found in patients with choledocholithiasis but without cholangitis [29]. Relatively atraumatic stone passage depends on two factors: stone size and OS diameter in its open state. Pharmacological agents such as aminophylline [10], theophylline [11], and anisodamine, a non-selective M-cholinergic antagonist [30], have been shown to increase the probability of spontaneous asymptomatic stone passage. However, the OS should not be damaged in order to prevent pancreatitis, which occurs more often with small bile stones [31,32]. While ERCP is the method of choice for the treatment of choledocholithiasis, it can cause complications; thus, pharmacological agents can be justified in order to avoid endoscopic interventions in some specific cases. In addition, isosorbide dinitrate has been used alongside balloon dilatation of the papilla to facilitate stone extraction without completely destroying the OS in order to reduce the risk of loss of sphincter function [33]. In our group, amylnitrit or sublingual nitroglycerin has been routinely used before ERCP [34].

A dynamic interaction exists between bacterial contamination of the biliary system, inflammation of the papilla and its eventual dysfunction, and the formation of bile stones [35]. Endoscopic and endosonographic alterations of the papilla have been described in cholelithiasis: an anisoecogenic papillary structure was detected in 48% of patients before cholecystectomy, and this proportion increased to 61% 3 months after the surgery and diminished to 25% after 6 months [36]. This suggests that gallstones and repeated passages of small fragments induce reversible changes in the papilla. The innervation of the OS also changes postcholecystectomy; CCK may no longer induce OS relaxation in a considerable proportion of these patients due to the loss of an inhibitory neural connection between the gallbladder and OS, potentially leading to paradoxical contraction due to the direct stimulatory effect of CCK on the OS smooth muscle [37].

#### 1.2.2. Biliary and Pancreatic Pain and OS Dyskinesia

**Figure 1:**
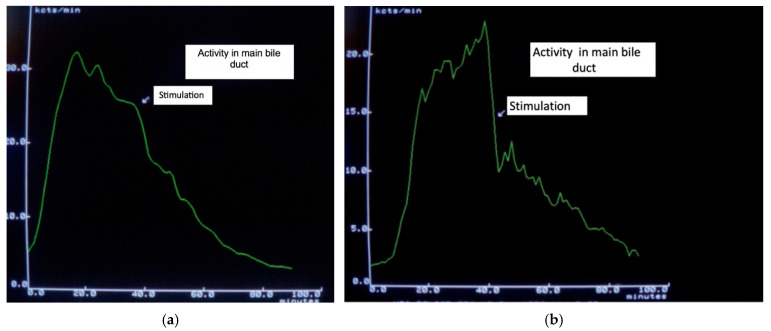
(**a**). Slightly accelerated transit after food stimulation. (**b**). Markedly delayed isotope transit after food stimulation, which had been normal previously, due to a partial functional obstruction.

Biliary and pancreatic pain remains the most controversial topic involving the OS. Several authors have even questioned the existence of functional OS disorders [18,38,39]. However, paradoxical contraction in response to a meal has been shown to cause temporary partial obstruction, which is visible in hepatobiliary scintigraphy (Figure 1).

Others advocate for the use of EST in suspected OS dyskinesia. The Milwaukee classification for OS dyskinesia [40,41] has been the most accepted standard for years. This classification distinguishes three grades of dyskinesis based on structural and functional anomalies: class I is equivalent to a stenosis, and it is considered a structural, organic disorder of the OS and not a functional disorder. In classes II and III, no organic disease of the papilla can be detected in the majority of cases. Manometry is considered the gold standard for diagnosing these two subclasses, though the diagnosis is difficult. The existence of class III OS dyskinesia is doubtful, as it is difficult to distinguish it from other functional intestinal disorders such as irritable bowel syndrome [42]. Thus, only class II meets the diagnostic criteria for a functional disorder of the OS. This has therapeutic management implications: class I requires endoscopic sphincterotomy without further diagnostic workup, while no intervention is recommended for class III SOD; these recommendations are supported by the EPISOD trials [43,44]. Class II, which is considered a unique functional disorder of the OS, requires individualized management and thorough patient assessment, which should include determining the intensity and frequency of painful events and the existence of eventual potentially life-threatening complications, such as recurrent pancreatitis. When discussing treatment, the patient should fully understand the benefits, risks, and alternatives to ERCP.

There are no data on the existence of progression of class II dyskinesis to organic OS stenosis. Class III OS dyskinesis treatment should also be individualized and consider every possible therapy used to treat functional digestive disorders.

#### 1.2.3. SOD and Postcholecystectomy Syndrome

While SOD can occur even in the presence of a gallbladder, it is far more common after cholecystectomy. The cholecysto-sphincteric reflex that participates in the postprandial physiological relaxation of OS is lost after cholecystectomy. Furthermore, an intact gallbladder acts as a “buffer”, reducing the bile duct pressure during an OS spasm. Adverse postcholecystectomy effects occurs in up to 47% of patients [45] manifesting as episodes of diarrhea, bloating, and abdominal pain. In murine models, permanently altered bile flow into the duodenum was found to disrupt the intestinal microbiota [46], leading to proinflammatory changes, which were exacerbated by a high-fat diet [47]. These changes could explain the symptoms reported by patients. The gut microbiota plays a crucial role in bile acid metabolism [48], which can create a vicious cycle.

Recurrent abdominal pain can occur after cholecystectomy. One explanation for this could be that the patient’s symptoms were not caused by the bile stones and thus the cholecystectomy did not resolve the original problem. Exhaustive diagnostic workup is necessary to exclude any other organic abdominal disease as well as postcholecystectomy complications, including residual bile duct stones. While magnetic resonance cholangiography (MRCP) is an excellent diagnostic tool, its diagnostic accuracy for smaller stones is lower [49]. Endoscopic ultrasound (EUS) has a considerably greater accuracy in the detection of small stones (microlithiasis) [50] and is also useful in the diagnosis of inflammatory changes or even tumors in the papilla. While endoscopic suspicion of papillitis is not infrequent, the diagnostic accuracy of EUS is superior: one study reported a sensitivity of 92.3% and specificity of 73.2% [51]. This is particularly important because papillitis is likely responsible for a substantial proportion of cases that were historically labeled as functional SOD. Indeed, already in 1995, Ponchon et al. reported that 30 of 69 papillary biopsies taken 10 days after EST treatment for SOD presented varying degrees of fibrosis and inflammatory infiltration [52]. These patients probably had rather an organic papillitis and not SOD. However, no evidence exists of a sequence of microlithiasis, repeated stone passage, papillitis and obstructive stenosis of the papilla.

Diagnostic workup must include MRCP, EUS, and duodenoscopy before considering a diagnosis of a functional disorder.

Once an organic disease and residual stones are excluded, we can consider functional disorders such functional dyspepsia, irritable bowel syndrome and, of course, SOD. Visceral hyperalgesia is a key factor in all of these disorders. Kurucsai et al. demonstrated hypersensitivity of the peripheral nociceptive nerve fibers in SOD patients without pain, but not in control individuals, after cholecystectomy [53]. Desautels et al. found that SOD class III patients exhibited duodenal-specific visceral hyperalgesia, and duodenal distention reproduced symptoms in all but one patient [54]. Notably, the Rome IV criteria [42] no longer recognize class III SOD as an independent entity distinct from other functional digestive disorders. Brawman Mintzer et al. [55] studied the psychosocial characteristics of the patients included in the EPISOD trial and found a high frequency of psychosocial comorbidities. However, this frequency was not significantly different compared with reported data in age-and gender-matched general populations. The brain–gut axis is a bidirectional connection between the nervous system and the gut, including the microbiota, that plays an important role in the symptoms of patients with functional gastrointestinal disorders, including SOD. Psychosocial factors interacts with GI function, motility, and visceral hyperalgesia to influence the intensity of patient symptoms [56].

Among functional disorders, SOD has great importance in clinical practice. It is frequently suspected, particularly when the patient’s pain is suggestive of a biliary origin or in cases of recurrent acute pancreatitis without other detectable etiologies. The Rome IV criteria [42] for a functional disorder of an epigastric or right upper abdominal quadrant pain are as follows:

1. Episodes lasting 30 min or longer.
2. Recurrent symptoms occurring at different intervals (not daily).
3. The pain builds up steadily.
4. The pain is moderate to severe enough to interrupt the patient’s daily activities or lead to an emergency department visit.
5. The pain is not relieved by bowel movements.
6. The pain is not relieved by postural changes.
7. The pain is not relieved by antacids.
8. Structural diseases that could explain the symptoms have been excluded.

Sphincter of Oddi dysfunction accounts for 1.8–31% [57] of cases in unselected populations with postcholecystectomy syndrome; this wide range illustrates the difficulties in objectively diagnosing this disorder. Indeed, in a preselected population that was likely to have SOD, the final diagnosis was only confirmed in 25–47% of cases [57]. The principal question is, how do we diagnose SOD or how can we exclude it? As mentioned above, EUS is the most sensitive method for detecting organic diseases of the biliopancreatic system, including microlithiasis, papillitis, tumors of the papilla of Vater, and early chronic pancreatitis, but not SOD.

## 2. Diagnostic Methods for Functional OS Disorders

Several methods have been described and used to diagnose OS dyskinesis. These methods induce OS contractions using morphine while simultaneously stimulating biliopancreatic secretion and monitoring the effects: pain, laboratory changes (including rapid elevation of hepatic and/or pancreatic enzymes), acute and transitory dilatation of pancreatic duct by magnetic resonance imaging, or prolonged bile duct transit time using isotope diagnostics. Non-invasive hepatobiliary scintigraphy, with or without food stimulation, and measurements of changes in pancreatic duct diameter after intravenous injection of secretin also can be used to detect abnormal OS function.

Another diagnostic approach is OS manometry, which was considered the gold standard for a long period but has been under reevaluation and discussion in recent years.

### 2.1. Choleretic-Morphine (Debray), Prostigmine–Morphine (Nardi), and Secretin–Morphine Tests

The simultaneous presence of OS spasms and the stimulation of the biliopancreatic secretion explains the postprandial pain in OS dyskinesis, though neither alone is sufficient to induce pain. Indeed, morphine and other opiates do not worsen acute pancreatitis (AP) [28]; once pancreatic inflammation is initiated, pancreatic secretion is dramatically decreased and does not respond to CCK stimulation [58], and even OS spasms cannot evoke increased pressure in the pancreatic duct system. However, both morphine [59] and codeine [60,61] can induce pancreatitis if pancreatic secretion is stimulated in healthy individuals. On the other hand, it is often difficult to distinguish abdominal pain of biliopancreatic or other origin. In this context, sublingual nitroglycerin can be administered: OS spasms will relax immediately with reduction or cessation of the pain, while pain of other origin does not respond.

Clinically positive results can be supported by significant and rapid increases in serum transaminase values in the Debray test [62], which may be accompanied by elevated serum amylase levels in the Nardi test [63,64] or in the secretin–morphine test. However, these tests lack specificity and there are doubts regarding their reproducibility [65]. In a previous study from our group [64], we demonstrated a good correlation between the results of Nardi and secretin–morphine tests and good reproducibility of the Nardi test in 17 patients. We also performed OS manometry in five patients with positive Nardi test results, four of them showed concordant results (unpublished data). In addition, repeatedly positive results of Nardi test became negative after endoscopic or surgical sphincteroplasty in four patients [64].

Visualization of partial bile flow obstruction is possible using hepatobiliary scintigraphy (HBS) [66,67], while only MR cholangiopancreatography or EUS can provide objective detection of transient increases in pancreatic duct diameter and fluid content in the duodenal lumen [68,69]. Several parameters have been evaluated using HBS, with hepatic hilum to duodenum transit time being the most sensitive. Timely changes in bile duct activity allow for the visualization of the dynamics of bile passage into the duodenum (see Figure 1). The initial publications reported a sensitivity close to 100% [70]; subsequent studies reported lower values but they remained relatively good. Hepatobiliary scintigraphy remains a useful method for detecting OS dysfunction with biliary manifestations, but not pancreatic involvement. However, a negative result does not definitively exclude SOD.

### 2.2. OS Manometry

OS pressure and motility can be studied using manometry, mainly via endoscopic cannulation of the biliopancreatic ducts. This method has long been considered the gold standard for diagnosing OS dyskinesia, with a basal pressure above 40 mmHg indicating functional disorder. The method has at least two disadvantages: it is invasive and may cause complications, particularly post-manometry acute pancreatitis, which occurs in 10–20% of cases [71]. Furthermore, pressure is only measured for few minutes and there are serious doubts regarding its reproducibility as repeated measurements may change from normal to pathological values or vice versa [72]. The advantage of this method is that it acquires objective numerical values. In our experiences, the frequency of pancreatitis can be considerably reduced using only two of the three lumens of the manometry catheter to record sphincter pressure, and aspirating bile or pancreatic juice through the third lumen. In our previous publication [73], no complications occurred when this method was used in 30 patients and only one case of post-manometry pancreatitis was observed among 100 cases. The use of electronic manometry catheters [74] may further reduce the pancreatitis risk. In some exceptional cases, the manometry result can help to determine if an endoscopic pancreatic sphincterotomy is needed to prevent recurrent pancreatitis and it can be used to measure reductions or even normalization of pancreatic sphincter pressure in parallel with clinical improvement [14]. Post-papillotomy manometry yielded an interesting observation: a paradoxical stimulatory effect of Buscapina on the OS motility [75]. We also confirmed the independent innervation of OS and duodenal muscle fibers: OS contractions were stimulated by Buscapina, while duodenal motility was consistently inhibited.

OS manometry has also been used to evaluate the long-term effect of EST complications in SOD. Several studies demonstrated reduced or normalized basal OS pressure, although elevated pressures were also reported [72]. Our unpublished results showed variable OS function after EST for bile duct stones: absent (n = 7) or markedly reduced (n = 8) basal pressure and contractile activity were observed in half of the patients studied, but the manometry results were normal in 10 patients and elevated pressures were found in four patients. In addition, in three of the seven patients who were presumed to have no sphincter, Buscapina produced paradoxical contractions, confirming the presence of residual muscular fibers [75]. Thus, endoscopic sphincterotomy—regardless of its original indication—does not completely destroy the OS in the majority of cases.

## 3. Who Needs Diagnostic Tests?

No further diagnostic methods are needed when the criteria for organic OS stenosis are met: fluctuating biliary type pain, variable but persistently abnormal hepatic laboratory values, and a dilated bile duct. Conversely, no diagnostic test should be performing on patients with diffuse abdominal complaints without objective alterations as the results would not affect therapeutic decisions. However, in these cases, personalized treatment for probable irritable bowel syndrome can be considered, such as trimebutine, which also relaxes the OS [76]. The patients who require additional diagnostic workup are former class II patients with pain accompanied by objective laboratory changes (elevated liver and/or pancreatic enzymes during pain episodes or recurrent pancreatitis) or mildly to moderately dilated biliopancreatic ducts. In this group of patients, we suggest performing a Nardi test, which can give information about biliary and pancreatic sphincter function, as well as imaging (hepatobiliary scintigraphy if dominant biliary dyskinesis is suspected or magnetic resonance cholangiopancreatography (MRCP) if recurrent pancreatitis is suspected). In our opinion, clinically negative test results without liver and pancreatic enzyme changes can reasonably exclude OS dyskinesia. In positive cases, individualized decisions should be made after a discussion with the patient and their family, weighing conservative treatment against endoscopic sphincterotomy [77] as it has limited utility and a considerably increased risk of post-sphincterotomy pancreatitis [78] and cholangitis [79].

As mentioned above, the specificity and sensitivity of these older diagnostic methods have been repeatedly questioned. However, we believe that with the Rome IV criteria and the findings from the EPISOD studies, the number of suspected SOD cases can be considerably reduced. Mild bile duct dilatation after cholecystectomy or slight elevations of transaminases in an overweight individual do not prove OS involvement. In our opinion, it is necessary to reconsider simultaneously performing provocation tests and hepatobiliary scintigraphy or MRCP.

### Recurrent Pancreatitis and OS

While the primary intracellular events in the pathophysiology of acute pancreatitis (AP) are widely known, the mechanisms underlying the intracellular calcium increase and proteolytic proenzyme activation remain unclear. One of the proposed mechanisms involves simultaneous pancreatic hypersecretion and outflow obstruction, in which OS plays a crucial regulatory role. Indeed, several cases of AP induced by morphine [41] or codeine [42,43] have been reported. Approximately 20% of recurrent pancreatitis cases are considered “idiopathic”; this proportion can be reduced to about 10–12% using endosonography (EUS), which can detect microlithiasis, early chronic pancreatitis, and pancreatic ductal anomalies. Indeed, ursodeoxycholic acid treatment resulted in perfect control of symptoms in 11 out of 12 patients with postcholecystectomy syndrome with microlithiasis [80]. Undetected microlithiasis is the most frequent cause of recurrent idiopathic pancreatitis when SOD is suspected. However, EUS cannot diagnose functional OS disorder [81]. In such cases, the non-invasive provocation tests described above could be useful and in positive cases, OS manometry can be performed to guide therapy. Pancreatic sphincterotomy is an invasive procedure and should be only performed if the expected benefits outweigh the risks [78,79]. Pancreatic sphincterotomy without demonstrated highly elevated basal pancreatic OS pressure will not provide benefits on the disease evolution. Similarly, although biliary sphincterotomy could reduce pancreatic OS pressure, it is also ineffective in the absence of OS dyskinesia.

## 4. Endoscopic Treatment for OS Dyskinesia

As described above, at present, only Milwaukee class II disorders meet the criteria for true functional SOD. Ren et al. [82] reported clinical improvement and normalization of laboratory parameters after EST and concluded that EST is a safe and effective treatment for SOD, even without prior manometry. However, in a subgroup of their patients with concomitant functional digestive disorders, 11 out of 12 experienced recurrent pain within 6 months. Roberts et al. [83] reported very comparable results and reached similar conclusions. Regarding RAP, endoscopic reintervention was required in 41.7% of patients [32]. In another group, serious adverse effects related to ERCP occurred in 68 out of 213 patients and at least one episode of recurrent AP was reported in group of 37 patients [84]. In a survey of endoscopists, the prevailing opinion was that SOD is a functional disorder that does not respond to endoscopic treatment [85]. A recent metanalysis [86] concluded that there is insufficient evidence to support the superiority of dual EST (biliary and pancreatic) compared with biliary EST alone, or even EST compared with sham interventions. Furthermore, comparative studies of EST and pharmacological treatment are lacking.

## 5. Pharmacological Treatment of OS Dyskinesia

Several drugs have been used to treat OS hypertonic dyskinesia. As described above, NO donors and calcium antagonists can produce rapid but transient OS relaxation and sublingual nitroglycerin can immediately relieve OS spasm-induced abdominal pain. However, long-term treatment with long-acting nitrates remains controversial due to the rapid development of nitrate tolerance [87,88]. Indeed, even in the treatment of coronary diseases, a nitrate-free period of at least 12 h was considered necessary to maintain the pharmacological effect.

An effect of trimebutine on OS dyskinesis was demonstrated by Barthet’s group [58]. As a widely used drug for the treatment of functional intestinal disorders with few and clinically insignificant side effects, trimebutine became the most used drug for the conservative treatment of SOD, often combined with sublingual nitroglycerin for acute pain [76,89]. EST was found to be more efficient in improving early symptoms in a group of SOD patients, but this superiority disappeared at later time points [89,90]. Duloxetine [91] and tricyclic antidepressants [92] have also been used with promising results.

Botulinum toxin injected into the papilla of Vater was also tested, with a positive response rate of approximately 70% [93], although the effects were transient and did not last longer than 6 months. This response has been used to select candidates for EST [94].

SOD management should always include recommendations for life-style changes, stress management, and the use of all available methods for treating other functional gastrointestinal disorders, such as mindfulness, yoga [95], and even acupuncture [96]. These mind–body interventions have proven to be an effective complement to more traditional therapies [97]. There are some extreme cases where spinal cord stimulation resulted in long-lasting pain relief and a return to normal daily life in patients whose disabling pain was refractory to all medical treatment attempts [98].

On the other hand, an acute obstruction, produced by a stone or OS spasm, is a very different situation. In this context, quick-acting nitrates (sublingual or i.v.) or theophylline can be used to treat functional spasms with excellent results. In addition, OS relaxation may facilitate uncomplicated passage of relatively smaller stones in some patients. These drugs have minimal side effects, so their administration can be recommended in these clinical situations in order to avoid invasive procedures.

## 6. Recommendations

**Figure 2:**
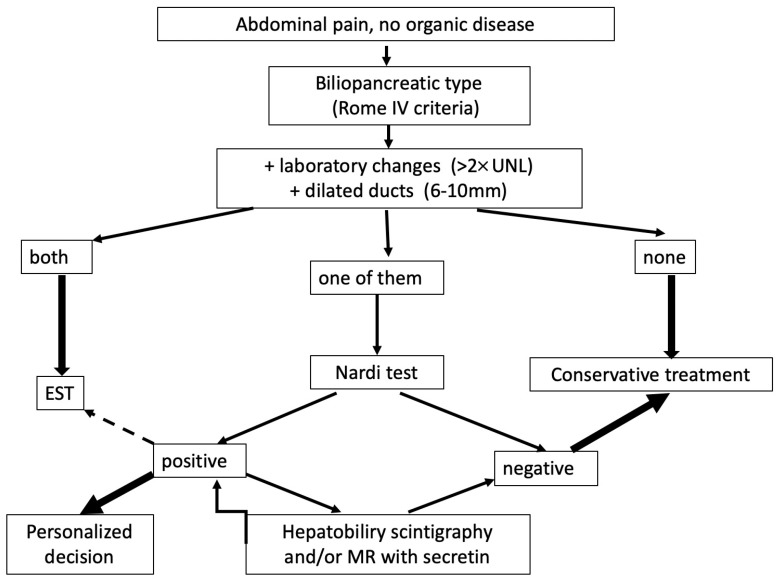
Procedure for diagnosing functional OS disorders.

Our proposed approach to diagnosing OS dyskinesis is summarized in Figure 2.

After excluding organic diseases using MRI, EUS, and duodenoscopy, the next step is to determine whether the pain is of biliopancreatic origin (typically postprandial pain occurring within 30 min, localized to the upper abdomen). If these symptoms are accompanied by elevated liver and/or pancreatic enzymes and ductal dilatation, it can be concluded to be an organic disease of the papilla of Vater and endoscopic treatment is recommended. If none of these parameters are present, significant OS disorder can be excluded, and further diagnostic workup is not necessary. The difficult diagnostic “gray” zone occurs when only one of these parameters is present. In such cases, a Nardi test is recommended, preferably simultaneously with the visualization of the bile duct (hepatobiliary scintigraphy) or pancreas (magnetic resonance imaging). A negative test result can exclude Oddi’s sphincter dysfunction with a high probability. Positive cases require individualized treatment, beginning with trimebutine and adding sublingual nitroglycerin for acute pain. High-risk ERCP and EST should be reserved for patients refractory to conservative treatment.

## 7. Conclusions

The OS plays a crucial role in the regulation of the outflow of biliopancreatic secretions. Damage or organic obstruction of the OS can be resolved using endoscopic sphincterotomy (EST). In contrast, a functional disorder of the OS, such as dyskinesia, is difficult to treat and should be managed conservatively in most cases, with EST reserved only for a minority of carefully selected patients.

## Data Availability

No new data were created or analyzed in this study. Data sharing is not applicable to this article.
